# Simultaneous Occurrence of Two Renal Artery Variations in a Single Potential Kidney Donor

**DOI:** 10.1155/crra/1016494

**Published:** 2026-01-17

**Authors:** Ampuriire Nyakubaho, Sematimba Henry, Ssempa Phiona, Ddumba Micheal, Michael G. Kawooya

**Affiliations:** ^1^ Department of Anatomy, Ernest Cook University (ECU), Kampala, Uganda; ^2^ Department of Radiology, Ernest Cook University (ECU), Kampala, Uganda

**Keywords:** accessory renal artery, angiography, case report, kidney donor, renal artery variation

## Abstract

**Background:**

Renal transplantation success depends on precise preoperative vascular mapping. This case report documents a rare instance of simultaneous dual renal artery variations in a prospective kidney donor.

**Procedure:**

A healthy 27‐year‐old male underwent MDCT renal angiography following standard protocols. The patient fasted for 8 h, received 80 mL of iohexol contrast at 1 mL/kg via a 20‐gauge cannula, and was imaged using a 32‐slice CT scanner. Scanning was performed from the descending aorta (just above the celiac trunk) to the iliac bifurcation using bolus tracking for optimal arterial enhancement.

**CT Findings:**

The CT scan demonstrated complex renal vascular anatomy. The right kidney was supplied by a main renal artery along with two accessory arteries, one originating laterally at L1 and another from the anterior aortic wall at L2. The left kidney exhibited a main renal artery with a single accessory branch arising immediately above its origin.

**Discussion:**

The embryological persistence of multiple aortic branches underlies these variations. Recognition of such anomalies is crucial as they may complicate donor nephrectomy through increased risk of intraoperative bleeding, complex vascular reconstructions, and compromised graft perfusion.

**Implications and Recommendations:**

Routine use of high‐resolution MDCT angiography is recommended for comprehensive donor evaluation. Enhanced interdisciplinary collaboration between radiologists and transplant surgeons is essential for tailoring surgical approaches and ensuring optimal outcomes.

**Conclusion:**

Detailed preoperative imaging is vital for identifying rare renal vascular anomalies, thereby optimizing surgical planning and promoting donor safety.

## 1. Background/Introduction

The success of renal transplantation heavily relies on a thorough understanding of the donor′s vascular anatomy. In recent years, multidetector computed tomography (MDCT) angiography has become the gold standard for preoperative evaluation, offering exceptional spatial resolution and three‐dimensional reconstructions of the renal vasculature [1]. Such precision is vital for identifying anatomical variants that could impact surgical planning, particularly in living kidney donors. In a systematic statistical meta‐analysis review of four online databases in accordance with PRISMA 2020 and evidence‐based anatomy work‐group guidelines on 111 studies, typical renal artery anatomy (a single bilateral vessel) was identified in 78.92%; the overall accessory renal artery prevalence was estimated at a pooled prevalence of 21.10% and estimated pooled prevalence of one, two, three, and four accessory renal arteries were 18.67%, 1.80%, 0.01%, and < 0.01% [2]. These vessels, remnants of the embryological arterial supply to the developing kidneys, generally pose no functional issues when present in isolation. However, the simultaneous occurrence of multiple renal arterial variations in a single individual is exceedingly rare as only one renal artery variation is commonly reported in every individual. In the case presented herein, the donor not only exhibited a left‐sided single accessory renal artery but also a right‐sided double accessory renal artery—an unusual combination that has been infrequently reported in the literature [3].

From a radiological perspective, the ability to accurately delineate such complex vascular anatomies is crucial. MDCT angiography not only facilitates the identification of these accessory vessels but also provides detailed insights into their origins, courses, and potential anastomoses. This level of detail is imperative for anticipating intraoperative complications such as unexpected hemorrhage or ischemic complications and post short‐ and long‐term operative challenges, which can arise if accessory arteries are not identified preoperatively [1, 4]. Moreover, understanding the embryological basis of these variations where multiple transient aortic branches persist due to incomplete regression during renal ascent further underscores the complexity encountered in certain donors. Given the surgical implications, the detection of dual renal artery variations necessitates an adjustment in the operative strategy to ensure optimal graft perfusion and minimize donor risk. It also serves as a reminder of the critical role of high‐resolution imaging in the modern era of transplantation medicine [4]. In this context, the case described not only enriches the existing body of radiological literature but also emphasizes the need for a meticulous preoperative vascular assessment in potential kidney donors.

## 2. Case Presentation

A healthy 27‐year‐old young man, a prospective kidney donor, was referred from Mulago National Referral Hospital in Kampala Uganda to the ECU Department of Radiology ECU for CT renal angiography.

### 2.1. Patient Preparation

The donor′s privacy and confidentiality were maintained throughout the procedure and was on nil per os for at least 8 h prior to the exam. Renal function tests were done to assess for glomerular filtration rate; urea and creatinine levels were normal. The donor was told to fill a written consent form after being explained the procedure, and he also reported no previous allergy to contrast media. The donor removed the clothes and radiopaque materials and was dressed in a patient gown, and his weight was measured, 80 kg. A 20‐gauge cannula was inserted in the patient′s arm, and an automatic injector was filled with 80 mL/s of iodinated contrast (iohexol) using a dosage of 1 ml per kg.

### 2.2. Donor Safety and Contrast Considerations

The safety of the potential donor during the imaging procedure is paramount. A critical component of the preprocedural protocol is the mitigation of contrast‐related adverse events. All donors undergo systematic screening for risk factors for contrast‐induced nephropathy (CIN), including assessment of baseline renal function via estimated glomerular filtration rate (eGFR). While the risk of CIN is low in healthy individuals with normal renal function, the use of low‐ or iso‐osmolar nonionic contrast media, such as iohexol, and adherence to weight‐based dosing are standard preventive measures [5]. Furthermore, the protocol includes screening for a history of contrast allergy, with contingency plans for premedication if necessary. These precautions ensure that the diagnostic benefits of MDCT angiography are achieved with minimal risk to the donor, aligning with the principle of primum non nocere in donor evaluation [6].

### 2.3. Client Positioning

The donor then lay supine head first with the head on a foam pad and the arms raised above the head and images were acquired during quiet breathing.

### 2.4. Equipment Used

A 32 slice Siemens Healthineers CT machine Somatom Go, 3rd generation was used, whereas injection was done using a single syringe medtron automatic injector pump.

### 2.5. Protocol Series

Under guidance of a radiologist, the imaging technologist supervised by a radiology resident used the renal angiography protocol.

## 3. Results

### 3.1. CT Findings of the Renal Arteries

The CT images were analyzed by a radiology resident and confirmed by a professor of radiology at the department.

### 3.2. Right Renal Arteries

There were three right renal arteries.

### 3.3. First Accessory Right Renal Artery

**Figure 1:**
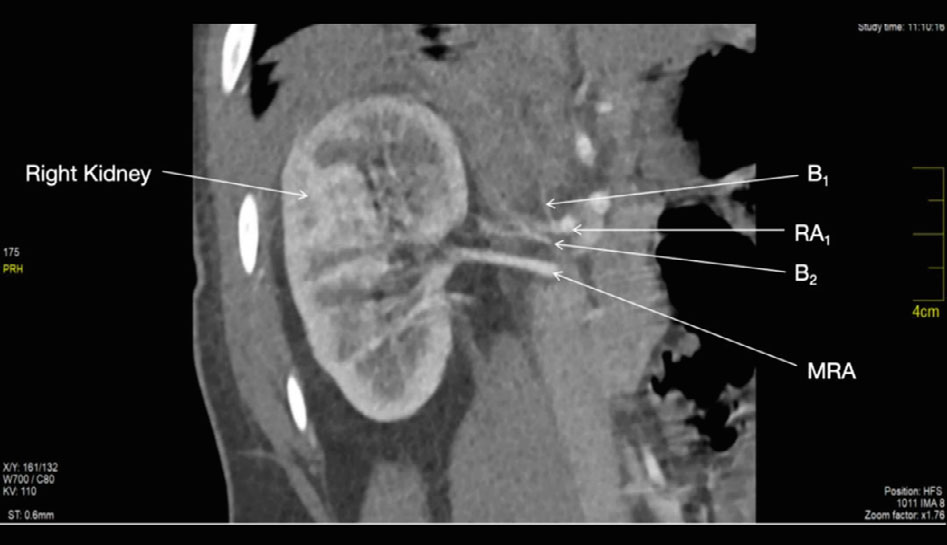
Showing the first right accessory renal artery and its first branch.

**Figure 2:**
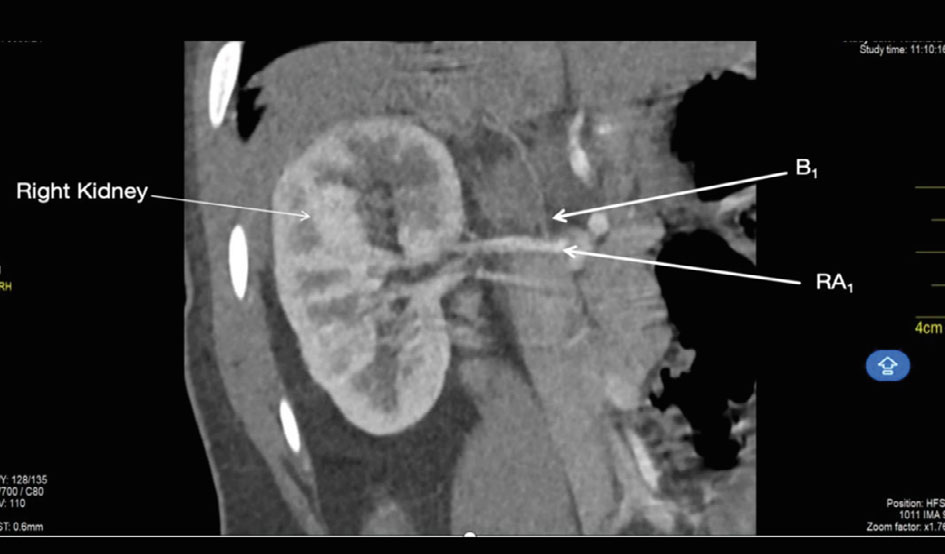
Showing the first right accessory renal artery, its branches and the right main renal artery.

The first accessory right renal artery, *RA_1_* originated from the right lateral border of the abdominal aorta at the level of the superior border of L1 (Figure 1). It coursed horizontally towards the hilum of the right renal hilum where it trifurcated to give off the upper, middle, and lower segmental arteries. It measured 0.40 cm at its maximum diameter. Two other vessels came off from the origin of this accessory artery. One of the vessels, B_1_, coursed superiorly toward the right hemidiaphragm. The second vessel B_2_ coursed inferior to the first right accessory renal artery for 1.24 cm before bifurcating to give off a branch that coursed toward the right adrenal gland and another branch that coursed toward the upper pole of the right kidney 9 (Figures 1 and 2).

### 3.4. Main Right Renal Artery (MRA)

The *MRA* originated from the right lateral border of the abdominal aorta at the level of the inferior border of L1, ~0.54 cm inferior to the origin of the first accessory right renal artery (Figure 2). It measured 0.47 cm at its maximum diameter. It briefly coursed inferiorly from its origin before running superolaterally toward the right renal hilum. At the hilum, it trifurcated to give off the upper, middle, and lower segmental arteries.

### 3.5. Second Accessory Right Renal Artery

**Figure 3:**
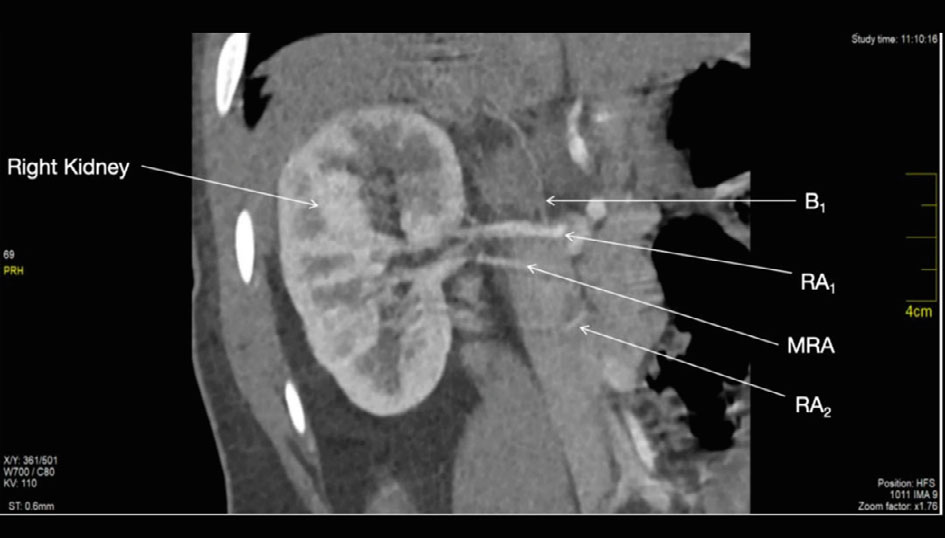
Showing the first right accessory renal artery, its branches, the right main renal artery and the second right accessory renal artery.

The second accessory right renal artery RA_2_ originated from the right anterior wall of the abdominal aorta at the level of the superior border of L2 (Figure 3). It measured 0.23 cm at its maximum diameter. It takes a horizontal but meandering course toward the lower pole of the right kidney. It trifurcated to give off segmental arteries before entering the right kidney.

### 3.6. Left Renal Arteries

There were two left renal arteries.

### 3.7. Left Main Renal Artery

**Figure 4:**
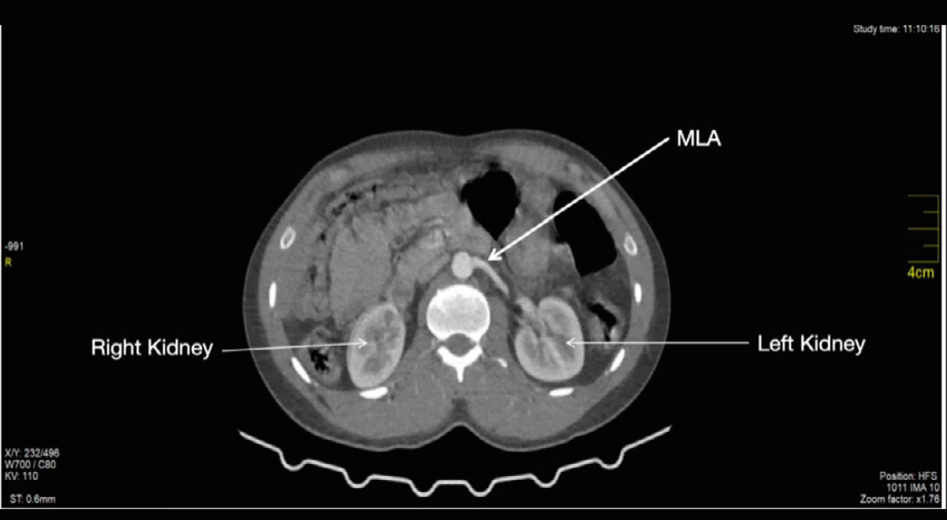
Showing the left main renal artery.

**Figure 5:**
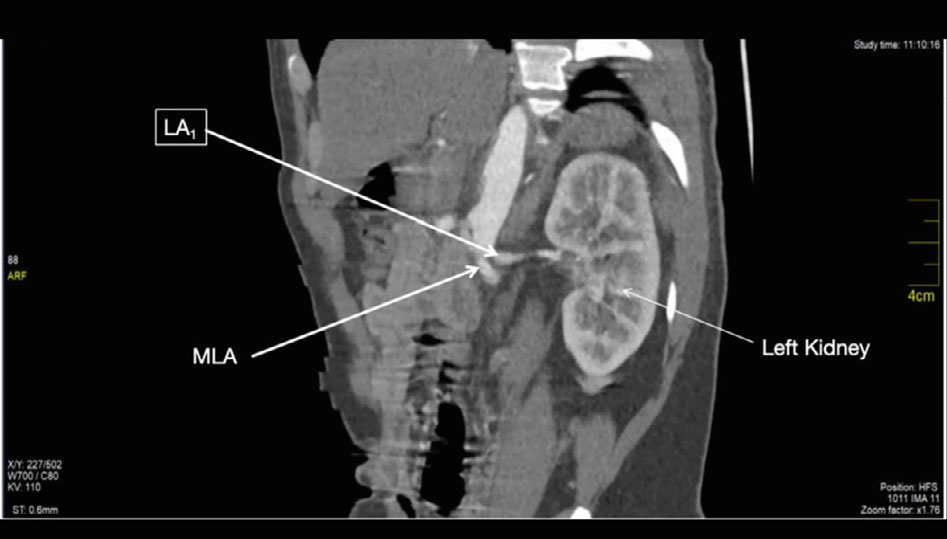
Showing the left main renal artery and the left accessory artery.

The main renal artery (MLA) arose from the left lateral wall of the abdominal aorta at the level of the lower border of L1 (Figures 4 and 5). It coursed inferiorly immediately after its take‐off before running horizontally towards the left renal hilum. It measured 0.55 cm at its maximum diameter. It gave off the first lower segmental branch ~2.83 cm from its origin. It then coursed 1.18 cm toward the hilum before bifurcating to give off the middle and upper lobe segmental arteries.

### 3.8. Accessory Left Renal Artery

The accessory renal artery (LA_1_) arose immediately above the origin of the main left renal artery (Figure 5). It measured 0.47 cm at its maximum diameter. It ran horizontally toward the left kidney and coursed almost parallel to the main left renal artery. It gave off the first branch ~0.93 cm from its origin; this branch coursed away from the hilum. It gave off another branch ~1.08 cm from the first branch; this branch coursed away from the hilum toward the upper pole of the left kidney. After the second branch, the accessory artery bent slightly inferiorly and coursed toward the hilum of the left kidney. At the hilum, it bifurcated to give off two segmental branches.

### 3.9. CT Conclusion on the Renal Arteries

1. Variant right renal arteries with a main renal artery and two accessory renal arteries.
2. Variant left renal arteries with a main renal artery and an accessory renal artery.

## 4. Discussion

Renal vascular anatomy is paramount in the preoperative evaluation of kidney donors, and the presence of accessory renal arteries demands careful attention to ensure surgical safety and optimal outcomes. In the present case, MDCT angiography revealed a rare configuration: a left‐sided single accessory renal artery combined with right‐sided double accessory renal arteries. This unusual presentation underscores the importance of high‐resolution imaging and a detailed anatomical understanding for preoperative planning [1, 7].

The genesis of renal arterial variations is intimately linked to embryological kidney development. During fetal growth, the kidneys ascend from the pelvic region and are initially supplied by several transient aortic branches. Normally, as the kidneys migrate, most of these vessels regress, leaving a single dominant renal artery per kidney. However, persistence of one or more of these embryonic vessels results in the formation of supernumerary or accessory renal arteries. In our case, the concurrent presence of a left accessory artery and two right accessory arteries likely reflects an atypical regression pattern influenced by genetic and hemodynamic factors during renal ascent [8]. These persistent vessels are not merely anatomical curiosities; they carry significant clinical implications in the context of kidney donation.

While the precise mechanisms behind the persistence of multiple renal arteries remain under investigation, several precipitating factors have been proposed. Variations in local expression of growth factors, hemodynamic stress during the critical period of renal migration, and genetic predispositions are thought to contribute to these anomalies. Such factors may alter the normal involution process of the transient arterial branches, leading to a higher incidence of accessory vessels in some individuals. Although accessory renal arteries are relatively common with prevalence rates estimated between 20% and 30%, the simultaneous occurrence of two distinct variations in a single donor, as observed in this case, is exceedingly rare and literature on its prevalence is scanty [7, 8].

Table 1 shows the tabulated summary of previously reported cases and series detailing rare double and triple renal artery variations in kidney donors. This comparison highlights the anatomical spectrum, surgical approaches, and outcomes associated with these variations.

**Table 1:** Author(s) and year, type of variation, donor type, number of cases, and key findings/outcome.

**Author(s) and year**	**Type of variation**	**Donor type**	**Number of cases**	**Key findings/outcome**
Damaskos et al. (2020)	Triple renal arteries (one main, two accessory)	Cadaveric	1 (left kidney)	Arteries preserved on a common aortic patch (Carrel patch); successful transplantation with normal graft function at 9‐year follow‐up.
Miclăuş et al. (2015)	Bilateral triple renal arteries (six arteries total)	Incidental finding (not a transplant donor)	1	Found incidentally on MDCT angiography; detailed measurements of arterial origin, length, and diameter provided. Highlights extreme anatomical rarity.
Panwar et al. (2020)	Double renal arteries with significant luminal discrepancy	Live‐related	58 recipients	Compared pantaloon anastomosis, end‐to‐side anastomosis, and separate implantation. Pantaloon technique feasible, safe, and associated with lower warm ischemia time.
Sarier et al. (2020)	Double, triple, and quadruple renal arteries	Living donors	2,144 donors (369 double, 35 triple, 2 quadruple)	CTA accurately detected variations: 17.2% double, 1.6% triple, 0.1% quadruple. Concordance between CTA and intraoperative findings was 97.9%.
Modi et al. (2024) (abstract only)	Double and triple renal arteries	Live donors	54 double, 2 triple (reported in series)	Retrospective study evaluating impact on graft function and outcome; details limited by access but confirms occurrence of triple arteries in live donors.

### 4.1. Transplant Board Decision and Impact of Preoperative Imaging

Following detailed MDCT angiography, the transplant board reviewed the complex vascular anatomy specifically the right‐sided double accessory renal arteries and the left‐sided single accessory artery and deemed the donor incompatible for kidney donation. The board concluded that the anticipated technical challenges, including increased risks of intraoperative bleeding, complex vascular reconstruction, and potential graft perfusion issues, outweighed the benefits of proceeding with nephrectomy. This decision highlights the critical role of high‐resolution preoperative imaging in clinical and surgical management: by precisely delineating anatomical variations, MDCT angiography not only guides surgical planning but also serves as a vital tool for risk stratification and donor selection. In this case, imaging directly prevented a potentially high‐risk procedure, prioritizing donor and recipient safety and reinforcing the importance of meticulous vascular assessment in transplant evaluation.

### 4.2. Implications for Kidney Donation

From a radiological perspective, the identification of multiple accessory renal arteries is crucial for surgical planning [9]. Despite the fact that *no intraoperative confirmation was available due to donor disqualification,* these variations can increase the complexity of donor nephrectomy by presenting challenges such as:

a. Intraoperative bleeding: Additional vessels may necessitate meticulous dissection and hemostasis to prevent significant blood loss.
b. Postsurgical complications: Accessory arteries to the lower pole may correlate with an increased rate of ureteral complications and a higher risk of complication and delayed graft function [10, 11].
c. Anastomotic complexity: The presence of multiple arteries might require complex reconstruction or multiple anastomoses during transplantation, potentially increasing operative time and the risk of vascular complications.
d. Graft perfusion: Ensuring adequate perfusion of the transplanted kidney is critical. Any missed accessory artery may compromise graft viability, leading to ischemic complications post‐transplantation.

MDCT angiography not only provides a three‐dimensional roadmap of the renal vasculature but also allows for precise localization of these accessory arteries, aiding in the anticipation and mitigation of surgical risks [1]. The radiological documentation of such variations plays an indispensable role in optimizing surgical outcomes and ensuring donor safety.

### 4.3. Recommendations

Based on the findings and the implications of the vascular anomalies, the following are recomended:

1. Routine use of MDCT angiography: All potential kidney donors should undergo comprehensive MDCT angiography to map out renal vascular anatomy accurately
2. Interdisciplinary collaboration: Radiologists and transplant surgeons must engage in detailed preoperative discussions to interpret the imaging findings and integrate them into the surgical strategy.
3. Tailored surgical approach: When multiple accessory arteries are identified, surgical planning should include strategies for vascular reconstruction or alternative approaches to ensure complete and safe graft harvesting.
4. Postoperative vigilance: Enhanced postoperative monitoring is recommended to promptly detect and address any vascular complications that may arise from these complex anatomical variations.

## 5. Conclusion

In conclusion, the present case illustrates a rare and complex renal vascular anomaly that has significant ramifications for donor nephrectomy. The simultaneous occurrence of a left accessory renal artery and right double accessory renal arteries is an uncommon finding that necessitates a high degree of vigilance during preoperative imaging. MDCT angiography proved to be an invaluable tool in delineating these variations, thereby guiding surgical planning and minimizing potential complications. The role of the radiologist is not only critical in identifying these anatomical nuances but also in clearly and precisely communicating their clinical significance to the surgical team. Such detailed vascular mapping is essential for the success of renal transplantation, ensuring both donor safety and optimal graft function.

**Nomenclature**
M.D.: medical doctor
Prof: professor
ECU: Ernest Cook University
eGFR: estimated glomerular filtration rate
CT: computed tomography
CTA: computed tomography angiography
CIN: contrast‐induced nephropathy
ROI: region of interest
HU: Hounsfield units
MIP: maximum intensity projections
RA: right accessory renal artery
MRA: main right renal artery
LA: left accessory renal artery
MLA: main left renal artery

## Ethics Statement

The authors have nothing to report.

## Consent

The participant was informed about the need to use the findings of the scan for learning, education and publishing purposes and consent was granted.

## Disclosure

This study complies with the CARE checklist attached below

## Conflicts of Interest

The authors declare no conflicts of interest.

## Author Contributions

Ampuriire Nyakubaho spearheaded the drafting of the whole manuscript and the acquisition of images for the publication. Sematimba Henry supervised the scanning process as well as the initial interpretation of the image findings to make the patient report. Ssempa Phiona & Ddumba Micheal aided in the patient preparation, planning and image acquisition. Michael G. Kawooya supervised the entire process and guided the review of the case report.

## Funding

No funding was received for this manuscript.

## Data Availability

The data that supports the findings of this case report are available from the corresponding author upon reasonable request
